# Multifunctional Nanostructures and Nanopocket Particles Fabricated by Nanoimprint Lithography

**DOI:** 10.3390/nano9121790

**Published:** 2019-12-16

**Authors:** Stefan Schrittwieser, Michael J. Haslinger, Tina Mitteramskogler, Michael Mühlberger, Astrit Shoshi, Hubert Brückl, Martin Bauch, Theodoros Dimopoulos, Barbara Schmid, Joerg Schotter

**Affiliations:** 1 AIT Austrian Institute of Technology, Molecular Diagnostics, 1210 Vienna, Austria; barbara-schmid@hotmail.com (B.S.); joerg.schotter@ait.ac.at (J.S.); 2 PROFACTOR GmbH, 4407 Steyr, Austria; michael.haslinger@profactor.at (M.J.H.); tina.mitteramskogler@profactor.at (T.M.); michael.muehlberger@profactor.at (M.M.); 3 Department for Integrated Sensor Systems, Danube University Krems, 2700 Wiener Neustadt, Austria; astrit.shoshi@donau-uni.ac.at (A.S.); hubert.brueckl@donau-uni.ac.at (H.B.); 4 AIT Austrian Institute of Technology, Photovoltaic Systems, 1210 Vienna, Austria; martinbauch1@gmx.de (M.B.); theodoros.dimopoulos@ait.ac.at (T.D.)

**Keywords:** nanoimprint lithography, nanostructure, nanocavity, nanoparticle, multifunctional nanoparticle, magnetic nanoparticle, plasmonic nanoparticle

## Abstract

Nanostructured surfaces and nanoparticles are already widely employed in many different fields of research, and there is an ever-growing demand for reliable, reproducible and scalable nanofabrication methods. This is especially valid for multifunctional nanomaterials with physical properties that are tailored for specific applications. Here, we report on the fabrication of two types of nanomaterials. Specifically, we present surfaces comprising a highly uniform array of elliptical pillars as well as nanoparticles with the shape of nanopockets, possessing nano-cavities. The structures are fabricated by nanoimprint lithography, physical and wet-chemical etching and sputter deposition of thin films of various materials to achieve a multifunctional nanomaterial with defined optical and magnetic properties. We show that the nanopockets can be transferred to solution, yielding a nanoparticle dispersion. All fabrication steps are carefully characterized by microscopic and optical methods. Additionally, we show optical simulation results that are in good agreement with the experimentally obtained data. Thus, this versatile method allows to fabricate nanomaterials with specific tailor-made physical properties that can be designed by modelling prior to the actual fabrication process. Finally, we discuss possible application areas of these nanomaterials, which range from biology and medicine to electronics, photovoltaics and photocatalysis.

## 1. Introduction

Nanostructured surfaces and nanoparticles are widely employed in many fields of research and technology, and there is an ever-growing demand for reliable and reproducible nanofabrication methods. In biology and medicine, nanostructured surfaces and nanoparticles are employed both because of their optical [1,2] or magnetic [3,4] properties alone as well as due to a combination of their optical and magnetic properties [5,6,7,8,9]. Nanostructured surfaces and nanoparticles are also employed in electronics, photovoltaics and sensing applications due to their specific physical properties [10,11,12]. Each application requires certain physical properties of the employed nanomaterial.

A huge variety of different nanomaterial fabrication methods can be found in literature [13,14,15,16,17]. In the present work, we apply nanoimprint lithography (NIL), which is a technique that uses a polymer resist into which a nanostructured stamp is pressed to transfer the nanostructure into the polymer. After resist hardening and stamp removal, a negative image of the nanostructure is obtained in the polymer. NIL was reported for the first time in the middle of the 1990s [18,19,20], and since then it was further developed and is now an established nanostructure fabrication method [21,22,23]. A specific type of NIL, called UV-NIL (ultraviolet nanoimprint lithography), is based on photocurable polymers and makes use of ultraviolet (UV) light to cure the polymer once the stamp is pressed into the latter [24,25].

Next to nanostructured surfaces, it was also shown that nanoparticles can be fabricated by combining NIL and physical vapor deposition or spin-coating techniques [26,27,28,29,30,31]. Multifunctional superparamagnetic and plasmonic nanoparticles were recently demonstrated by Zhang et al. [32], who employed a spin-coating process of colloidal nanocrystals onto an imprinted surface, which finally, after a ligand exchange step, resulted in the fabrication of rod-shaped nanoparticles.

In the present study we show that UV-NIL can be used to fabricate an array of nanoparticles, starting from a uniform array of elliptical pillars, and that these nanoparticles can be transferred to solution yielding a nanoparticle dispersion. The so fabricated nanoparticles have the shape of nanopockets as each of them possesses a cavity. Furthermore, multifunctional optical and magnetic properties of the nanopockets were obtained by incorporating different materials. Scanning electron and scanning force microscopy, as well as optical transmittance measurements give experimental evidence of the successful nanofabrication. Finally, we also show optical simulation results which are in good agreement with the experimentally obtained data. This also means that the optical nanopocket properties can be tailored to specific demands by predictive modelling prior to the fabrication process. In conclusion, we present a highly versatile nanofabrication method that allows nanomaterials to be produced for a wide range of applications, which are discussed in more detail in the final section.

## 2. Materials and Methods

**Figure 1:**
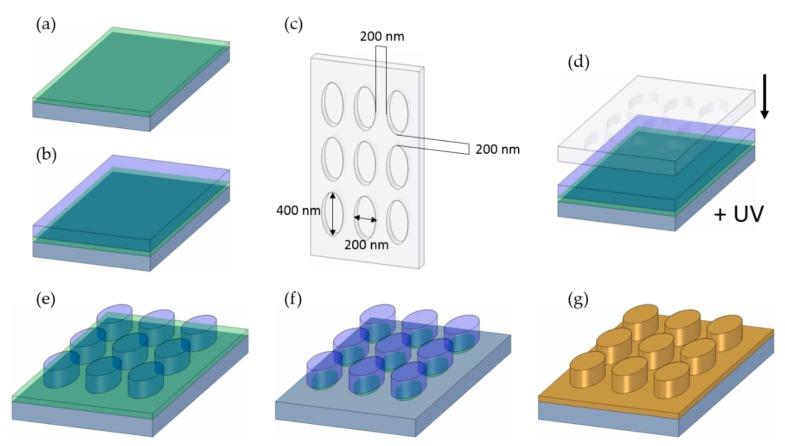
Schematic nanostructure fabrication (not to scale): (**a**) deposition of the lift-off resist (green) on top of a silicon substrate; (**b**) deposition of the imprint resist (blue); (**c**) employed stamp with elliptical holes separated by a gap of 200 nm as indicated and elliptical holes with a major axis of 400 nm and a minor axis of 200 nm; (**d**) actual imprint process of the imprint resist by pressing the stamp into the resist, followed by curing the resist by ultraviolet (UV)-light exposure; (**e**) imprinted structure in the resist after removal of the stamp with a pillar height corresponding to the imprint resist thickness of 200 nm; (**f**) final imprint structure after reactive ion etching and wet-chemical etching, resulting in an undercut etch of the lift-off resist; (**g**) deposition of a Au layer of 50 nm thickness to obtain the final metal nanostructure.

The custom silicon UV-NIL master was commercially purchased (IMS Chips, Stuttgart, Germany) and treated with an anti-sticking layer (BGL-GZ-83 produced by PROFACTOR GmbH, Steyr, Austria). The master shows a regular pattern of elliptical pillars. The pillars have a height of 250 nm and an elliptical base with a major axis of 400 nm and a minor axis of 200 nm. The pillars are separated by a gap distance of 200 nm from each other. The so structured area extends over 1 × 1 cm^2^ and contains about 4.2 × 10^8^ pillars. From the master, a hybrid stamp composed of polydimethylsiloxane (PDMS) and hard PDMS (h-PDMS) was fabricated by methods reported in literature (see also Figure 1c for a schematic representation of the stamp) [33,34]. The process of the UV-NIL imprint with an inverse structure was in parts presented recently [35,36].

Silicon wafers with a thermally grown oxide layer of 300 nm thickness served as sample substrate. Spin-coating processes were performed on samples at room temperature. The lift-off layer of polydimethylglutarimide-based resist (LOR 1A obtained from micro resist technology GmbH, Berlin, Germany) was spin-coated at 4000 rpm (100 nm film thickness) and hard baked at 200 °C for 5 min on a hotplate. The organic UV-curable imprint resist layer (mr-NIL212FC_XP purchased from micro resist technology GmbH, Berlin, Germany) was spin-coated at 4000 rpm (200 nm film thickness) and soft baked at 100 °C for 1 min. The imprint was carried out by pressing the h-PDMS/PDMS stamp into the imprint resist and curing with a self-built high-power UV-LED lamp (30 mW/cm^2^ for 1 min at a wavelength of 365 nm). The stamp was removed, and the imprinted sample placed on the hotplate for a hard bake process at 150 °C for 1.5 min. The following reactive ion etching was performed in an atmosphere of 15 at% O_2_ at a pressure of 0.24 mbar for 2 min to remove the residual layer of uncured resist. The wet-chemical etching process was done with a 1:1 dilution of a metal ion free developer (MICROPOSIT MF-24A obtained from micro resist technology GmbH, Berlin, Germany) in ultra-pure water and an immersion time of the sample of 30 s at room temperature. The sample was then thoroughly rinsed with ultra-pure water and dried under a nitrogen stream (see Figure 1f for a schematic representation of the fabricated nanostructured surface).

The deposited layers were fabricated by DC magnetron sputtering (UNIVEX 450C from Leybold Systems, Cologne, Germany) with a target to substrate distance of about 100 mm. Au and NiFe (Ni:Fe 80:20 at%) were sputtered in pure Ar atmosphere at a gas pressure of 2 µbar and at a power of 20 W and 40 W, respectively. TiO*x* was sputtered from a Ti target by a reactive sputter process in an Ar:O_2_ 80:20 at% atmosphere at a gas pressure of 1 µbar and a power of 120 W. The sputter rates of Au, NiFe, and TiO*x* were 0.548 nm/s, 0.141 nm/s, and 0.014 nm/s, respectively.

For the transfer of the nanopocket array from an opening-down to an opening-up configuration, three different commercially available adhesive tapes were employed. One tape was applied to allow for sputter deposition and electron microscopy due to its vacuum stability (polyimide tape, article number 5413 from 3M^TM^). The second was applied for transmittance measurements due to its transparency (article number 56002 from TIXO^®^). The third one was applied for a transfer of the nanopockets into solution due to its water solubility (water soluble wave solder tape, article number 5414 from 3M^TM^).

The transfer of the nanopockets into solution was achieved by ripping-off the nanopockets from the substrate with the water-soluble tape, followed by dissolving the latter in water at 45 °C. To remove the remains of the dissolved tape and to wash the solution, we placed a magnet next to the sample vial to collect the particles and replaced the fluid by fresh ultra-pure water (18.2 MΩ cm resistivity). This process was repeated three times.

Scanning electron microscopy (SEM) images were recorded on a Zeiss Supra 40 electron microscope at 5 kV acceleration voltage using the in-lens detector. The nanostructures on the Si substrate were imaged directly on the substrate. The images of the opening-up nanopocket array were taken after sputter deposition of a 20 nm Au layer for the generation of a conductive top layer to allow for SEM. The nanopockets from the solution were prepared for imaging by drop-casting onto a Si wafer substrate with a native oxide surface and letting the solution dry at room temperature.

The sample surface topography was characterized by scanning force microscopy (SFM) (Molecular Imaging, Pico Plus) in acoustic mode with PPP-NHCR tips (NANOSENSORS^TM^).

Optical transmittance of the obtained nanostructured layers was measured by a Bruker Vertex 70 Fourier transform infrared spectrometer (Bruker Corporation, Billerica, MA, USA) equipped with an additional visible light source and a Si photodetector. For polarization control of the incident light beam, a linear polarizer was inserted in the light path (wire grid polarizer with article number WP25M-UB from Thorlabs GmbH, Newton, NJ, USA). The measurements were recorded at an incident light beam normal to the sample surface and referenced to the substrate material. The transmittance curves obtained were smoothed and plotted using commercially available software from OriginLab Corporation, Northampton, MA, USA.

Optical simulations were conducted using a commercial software based on the finite-difference time-domain (FDTD) method (FDTD Solutions, Version 8.7.4 from Lumerical Inc., Vancouver, BC, Canada). The complex refractive indices of Au and glass were taken from literature [37,38]. Transmittance simulations of the periodic nanopocket array, as well as electric field maps were simulated for a single unit cell with periodic boundary conditions, for normal incident light and air as medium. A broad band plane wave light source was assumed for the simulations. The mesh size for the simulation was uniform in all three spatial directions with 1 nm for the case of polarization parallel to the short ellipse axis and 5 nm for polarization parallel to the long ellipse axis. The electric field maps were normalized to the incident electric field magnitude.

The magnetic moment was measured by a Lake Shore Cryotronics Inc Westerville, OH, USA 8600 series vibrating sample magnetometer (VSM). The nanopockets were drop-casted from aqueous solution onto a silicon wafer substrate and let dry resulting in a random nanopocket orientation. Measurements were carried out with the external magnetic field oriented in-plane of the substrate at room temperature.

## 3. Results

The nanostructure fabrication process is schematically shown in Figure 1. It started by spin-coating a lift-off layer with a thickness of 100 nm on top of the silicon substrate (Figure 1a). On top of this layer, we deposited the UV-curable imprint layer with a thickness of 200 nm (Figure 1b). Next, the actual imprint was performed by pressing a custom fabricated stamp (Figure 1c) into the imprint layer, followed by UV-curing of the latter (Figure 1d). As a result, a negative copy of the stamp nanostructure was fabricated on the substrate in the imprint layer after removal of the stamp (Figure 1e). After the imprint process, a thin residual imprint resist layer remained on top of the surface. This residual layer and most of the underlying lift-off resist layer were removed by a reactive ion etching step. An additional hard bake process after the imprint ensured that the etch rate of the imprint layer was lower than that of the lift-off layer. To fully remove the lift-off layer in between the pillars, we performed an additional wet-chemical etching step, which selectively targeted the lift-off layer. The latter also resulted in an undercut etching of the lift-off layer. The result of these fabrication processes is shown in Figure 1f. Finally, we fabricated a gold metal film of 50 nm thickness onto the sample by sputter deposition, which is a mainly isotropic coating process that results in the coverage of the whole sample surface (Figure 1g).

**Figure 2:**
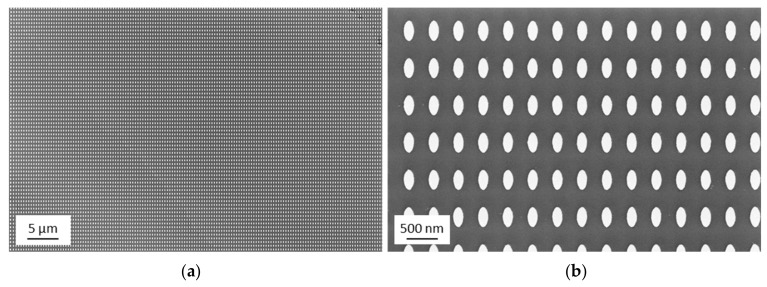
Final ultraviolet nanoimprint lithography (UV-NIL)-structured surface. Scanning electron microscope (SEM) after the imprint and the etching processes: (**a**) overview SEM image; (**b**) detailed SEM image.

In the following, we will give experimental evidence of the nanostructures fabricated by UV-NIL and sputter deposition (see schematics in Figure 1f,g). SEM images of the final UV-NIL imprinted sample are presented in Figure 2 (corresponding to the schematic shown in Figure 1f). An overview with lower magnification (about 10,000 pillars) as well as a more detailed image with higher magnification are given. The image shows that the structures have a high degree of uniformity. This accounts for the whole imprinted area of 1 × 1 cm^2^. With an inverse imprint structure of the same master, it was recently shown that a very narrow size variation of the imprinted structures is achieved, resulting in a standard deviation of the area of the elliptical base of +/−3% [35].

**Figure 3:**
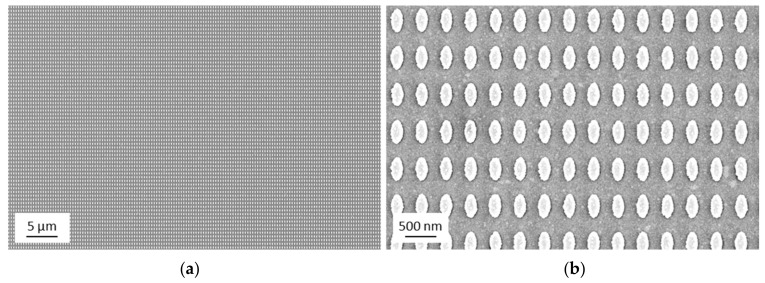
Nanostructure array after the sputter process resulting in a Au layer of 50 nm thickness: (**a**) overview SEM image; (**b**) detailed SEM image.

Next, the sample was coated by sputter deposition with a Au film of 50 nm thickness. SEM images of the resulting nanostructure are shown in Figure 3. Again, an overview with lower magnification (about 10,000 pillars) as well as a more detailed image are presented. The high degree of uniformity of the pillar array also remains after the deposition of the Au layer. The morphology of the thin gold film grown by sputter deposition results in a grain-like structure on the top surface and corresponds to gold film growth reported in the literature [39].

**Figure 4:**
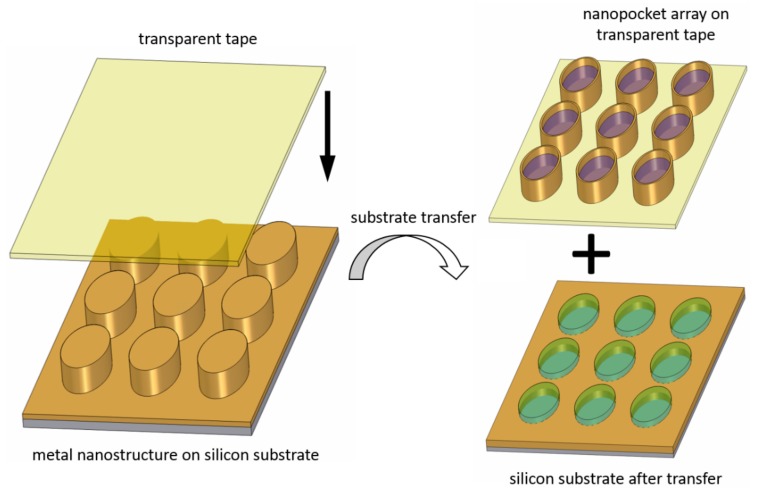
Schematic sketch of the substrate transfer from solid silicon to a transparent substrate. On removal of the transparent substrate, nanopockets are created. While the imprint resist stays inside the nanopockets, the lift-off resist remains on the original substrate.

The elliptical pillars of the metal nanostructure were then mechanically transferred from the Si substrate onto a piece of tape, which resulted in an array of nanopockets facing their open entities towards the top (a schematic sketch is shown in Figure 4). Here, the metal layer in between the pillars remains on the original substrate so that the metal nanopockets are separated from each other (i.e., not connected by the Au metal anymore). In other words, the nanopockets are transferred from an opening-up to an opening-down configuration.

**Figure 5:**
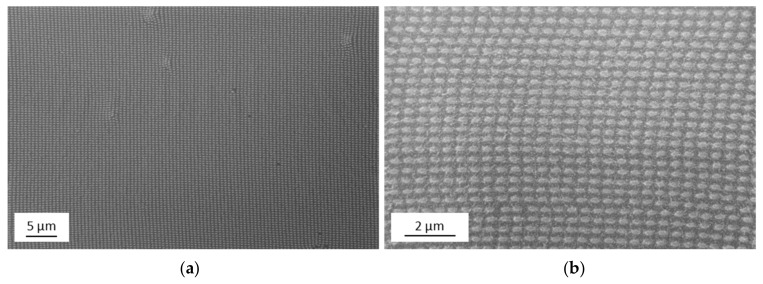
SEM imaging of the opening-up nanopocket array on the polyimide tape after depositing a 20 nm thick Au layer on top of the structure for sample preparation: (**a**) overview SEM image; (**b**) detailed SEM image.

The transfer of the nanopockets was achieved by using commercially available tapes that were either transparent or made of polyimide. The successful transfer of the pillars also indicates that the sputter deposition process is not completely isotropic. The metal film at the side walls is thinner towards the bottom of the pillars, especially at the location of the lift-off resist undercut, where the metal film is very thin or even broken. SEM images of the transferred nanopocket array on the polyimide tape are shown in Figure 5, where Figure 5a shows about 10,000 nanopockets. To allow for electron microscopy imaging, we sputter-deposited an additional layer of 20 nm Au on top of the entire structure to create an electrically conductive sample surface. It can be concluded that the nanopocket array stays intact and is almost perfectly transferred. Ripples can be seen in Figure 5a, which originate from the flexibility of the employed tape.

**Figure 6:**
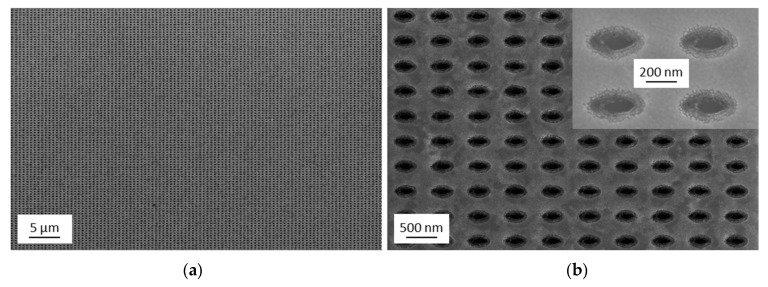
SEM imaging of the substrate after the tape transfer step: (**a**) overview SEM image; (**b**) detailed SEM image and a high magnification image of four of the transferred pillars in the insert on the top right.

The uniform transfer of the nanopockets to an opening-up configuration over the wide sample surface was also verified by examination of the substrates after the tape transfer step. SEM images of the substrate after the transfer are shown in Figure 6 with about 10,000 removed nanopockets. It was observed that the nanopockets are very efficiently and uniformly removed from the substrate. Furthermore, it can be concluded from the SEM images that the Au layer in between the pillars remains on the substrate and is not transferred.

**Figure 7:**
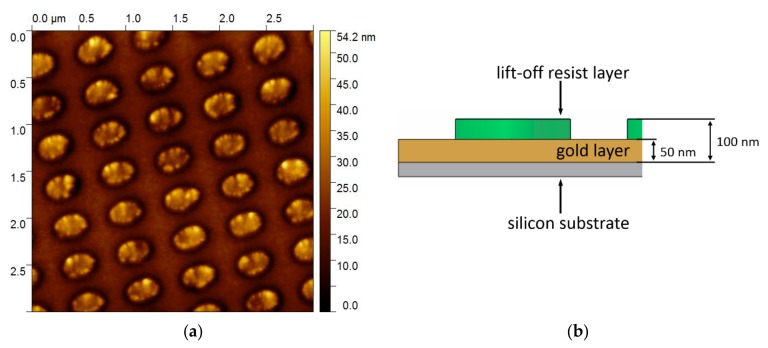
Scanning force microscopy (SFM) image of the substrate after the tape transfer process and schematic sketch of the layer thicknesses. (**a**) The remaining elliptical pillars can be observed by SFM imaging. The height of the imaged structure is indicated by the color bar at the right of the SFM image. (**b**) Schematic sketch of the cross section of the sample indicating the layer thicknesses.

The substrate after the tape transfer was further characterized by SFM imaging, which is shown in Figure 7a. The elliptical resist pillars partly remain on the substrate, so it can be concluded that the nanopockets have a hollow entity. The depth of the hollow entity corresponds to the height of the remaining resist pillars and amounts to about 50 nm as determined by SFM. The thickness of the lift-off resist amounts to 100 nm and the Au layer has a thickness of 50 nm. Together with the remaining 50 nm pillar height, this means that the resist pillars are broken at the interface of the lift-off and the imprint resist (see Figure 7b for a schematic sketch of the layer thicknesses). The imprint resist stayed inside the nanopocket and the lift-off resist remained on the original substrate. The hollow entity of the nanopockets consequently has a depth of 50 nm. This implies that the depth of the hollow entity can be adjusted via the lift-off resist thickness and the thickness of the deposited layer by modifying the spin-coating parameters. It is advantageous that the UV-curable imprint resist is not sensitive to the wet-chemical etching once it has been hardened by UV light. This allows to etch the underlying lift-off resist only and can also be done after the curing of the imprint resist.

**Figure 8:**
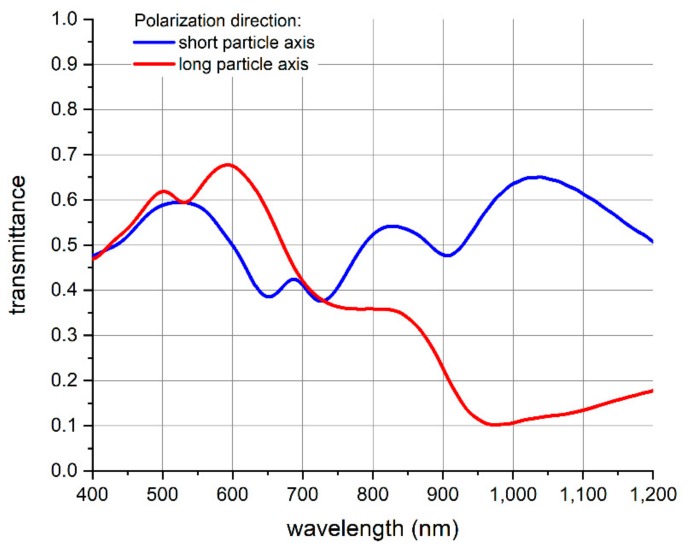
Optical transmittance spectrum measurements of the nanopocket array on a transparent film. The transmittance was measured in dependence of the polarization direction of the linearly polarized incident light beam. Polarization of the incident light parallel to the short ellipse axis corresponds to the blue line, while the red line is the measured transmittance for the case of polarization aligned parallel to the long ellipse axis.

To further characterize the transferred nanopocket array, we did a tape transfer step using a transparent tape. Thereby, we measured the optical transmittance of the nanopocket array as a function of the wavelength in the visible and near-infrared regime. This characterization was further undertaken in dependence of the polarization direction of the incident light beam (see Figure 8). The latter was linearly polarized with a polarization axis aligned parallel as well as perpendicular to the long axis of the ellipsoidal nanopocket.

**Figure 9:**
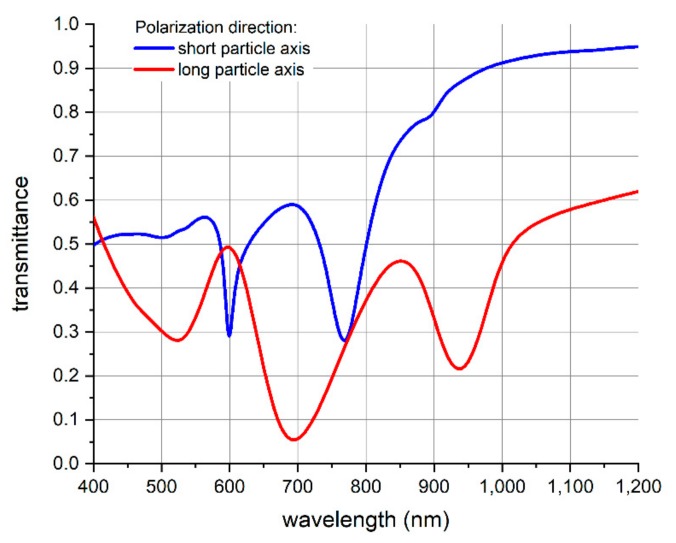
Optical simulation results of the transmittance spectrum of the nanopocket array on a glass substrate. The transmittance is plotted in dependence of the polarization direction of the incident light beam. The blue line shows the transmittance for incident light polarized along the short ellipse axis and the red line shows the transmittance for polarization along the long ellipse axis.

Furthermore, we conducted optical simulations of the wavelength-dependent transmittance of a nanopocket array, which are shown in Figure 9. To guarantee simulation results that are best-comparable to the experimental values, we have chosen the nanopocket dimensions as determined by SEM (see also Figure 12). Regarding the outer dimensions, the ellipse minor axis amounts to 170 nm, the major axis to 380 nm and the height of the nanopocket was 250 nm. The gold layer thickness is 50 nm. These nanopockets are arranged as an array corresponding to the imprint stamp. The substrate is glass while the surrounding medium is air. The schematic geometry applied for the simulation corresponds to what is shown on the right-hand side in Figure 4.

Table 1 shows a comparison of the minima peak positions of the experimentally measured and the simulated results. Clearly, there is good correlation between the spectral position of the measured and the simulated transmittance minima. The minor deviation of the simulated values from the measured values can be explained by the actual morphology of the nanopockets in comparison to the smooth and uniform geometry assumed for the simulation. Furthermore, we simulated the nanopocket array on a glass substrate with air as surrounding medium, while the experimentally applied nanopocket film was an array of nanopockets embedded in the glue film of the tape with the latter as substrate.

**Figure 10:**
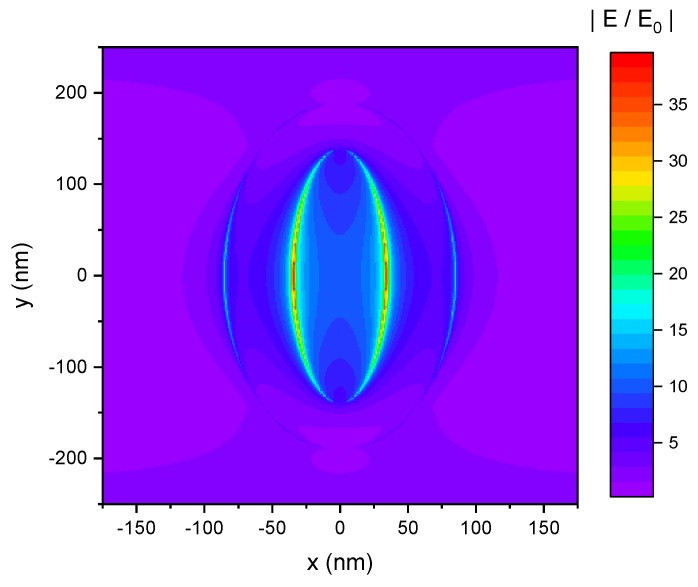
Electric field map of a single nanopocket. Electric field enhancement |E/E_0_| with respect to the incident electric field E_0_ at the top surface of the nanopocket. The polarization direction is along the short ellipse axis.

Based on the simulations of the transmittance, we also conducted simulations of the enhancement of the electric field |E/E_0_| with respect to the incident electric field E_0_ for incident light polarized along the short ellipse axis. A corresponding electric field map is shown in Figure 10 and shows the electric field enhancement at the top upper rim of the nanopocket at a wavelength of 768 nm, i.e., the second minimum position of the transmittance. It can be seen that the electric field is enhanced by up to a factor of 40. This resonant excitation occurs at the upper rim inside the cavity of the nanopocket. The electric field enhancement increases with decreasing size of the cavity (decreased distance between two opposite gold faces). It can also be observed that the electric field is enhanced inside the whole cavity.

Summarizing the simulation results, good correlation of the experimental and the simulated values was obtained. Together with the flexible fabrication method, this indicates that the optical characteristics of the nanopockets can be tuned to application demands, e.g., resonance at a certain wavelength, which also includes electric field enhancement.

**Figure 11:**
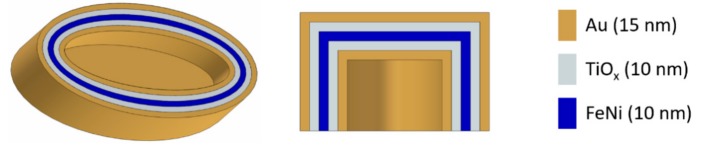
Schematic representation of a nanopocket composed of three different materials. The whole nanopocket is shown on the left, while a vertical cut along the short ellipse axis is shown in the middle. The employed materials are sketched on the right.

Obviously, the fabrication of the layer via sputter deposition allows other materials to be used as well. We fabricated nanopockets with a three-layer stack of 15 nm Au, 10 nm NiFe, and 10 nm TiO*x* to show the versatility of the fabrication method, leading to multifunctional nanopocket structures. While gold is commonly employed for optical reasons [40,41], NiFe serves as a magnetic layer to realize magnetic nanoparticles [4,14], and the TiO*x* can be used as a dielectric layer which can, for example, be interesting for energy conversion and photochemistry applications [42,43]. In the chosen multilayer structure, the TiO*x* also electrically insulates the magnetic from the gold layer. We have chosen a symmetrical composition of the single materials so that the material that is facing the surrounding medium is gold. This is advantageous for later applications where the nanopockets need to be stabilized in solution. Additional linker molecules can be easily bound to the gold layer by employing well-established thiol-linker chemistry. Figure 11 schematically shows the fabricated nanopocket.

**Figure 12:**
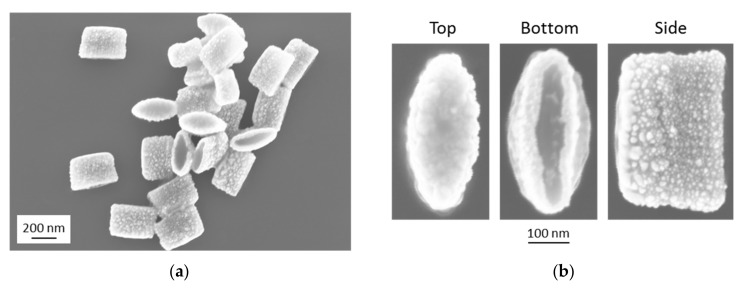
SEM images of the fabricated three-material multifunctional nanopockets: (**a**) overview SEM image of randomly oriented nanopockets; (**b**) detailed SEM image with higher magnification of the top, bottom and side view of a nanopocket.

After the sputter-deposition processes, we ripped off the nanopockets from the substrate by a water-soluble tape. The latter was then dissolved in water. Due to the magnetic layer, we were able to conduct magnetic washing and separation steps by placing a permanent magnet next to the sample vial. This allowed residues of the tape to be removed and a transfer of the nanopockets into ultra-pure water. SEM images of these nanopockets are shown in Figure 12 after drop-casting them from solution onto a silicon wafer substrate and letting the drop dry at room temperature. While Figure 12a shows the nanopockets as they were imaged in a random orientation after the drying process on the substrate, Figure 12b shows the top, bottom and side view of such a nanopocket. The typical grain-like growth of the Au can be identified clearly.

**Figure 13:**
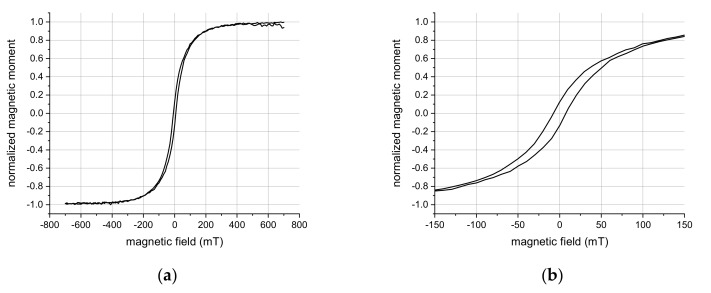
Vibrating sample magnetometer (VSM) measurements of the fabricated three-material multifunctional nanopockets: (**a**) obtained hysteresis loop with magnetic field strengths that saturate the magnetic moment of the nanopockets; (**b**) zoom into the hysteresis loop.

While the magnetic washing steps already indicated a successful deposition of a magnetic layer, we also characterized the magnetic properties by measurements of the magnetic moment in dependence of an externally applied magnetic field. This was accomplished by recording the hysteresis loop of drop-casted nanopockets on a silicon wafer substrate by a VSM. Figure 13 shows the normalized magnetic moment, which saturates above 400 mT, and a zoom into the hysteresis loop. The nanopockets show a weak ferromagnetic behavior with a coercive field of about 6.5 mT and a clear remanence.

## 4. Discussion and Conclusions

Our results show that a complex nanostructure composed of an array of pillars with elliptical base can be fabricated by UV-NIL and sputter deposition. This uniform nanostructure array expands over a large sample area of 1 × 1 cm^2^. Additionally, we could show that this nanostructure array can be flipped upside-down and transferred onto another substrate without affecting the uniformity of the array. It was shown that the so transferred nanostructure is an array of single nanoparticles separated from each other. These nanoparticles have the shape of nanopockets as each of them possesses a nano-sized cavity on one side. The depth of this cavity can be controlled by the applied resist layer thicknesses and the thickness of the deposited layer. Nanostructures and nanopockets made of a single Au layer were presented, but it was also shown that the flexibility of the fabrication method allows multifunctional nanopockets to be created that can be dispersed in solution. For example, we fabricated nanopockets composed of three different materials, i.e., Au, NiFe and TiO*x*. This implies that the functionality of the nanopockets can be tailored to meet specific demands. We have already shown that the magnetic properties of the nanopockets, resulting from their NiFe layer, can be exploited to execute magnetic washing and separation steps of a nanopocket dispersion. Next to the flexible choice of employed materials, the nanoparticle geometry can also be easily adjusted by the layout of the initial master and the different resist film thicknesses in the nanoimprint process. Finally, the fabrication techniques employed are well-suited for upscaling as documented for NIL [44,45]. Upscaling can also be supported by commercially available inline sputter deposition systems.

Additionally, our simulation results are in good agreement with the experimentally obtained data, which indicates that desired specific physical properties of the nanopockets can be experimentally achieved after an initial modelling step. By all these measures, it will be possible to tailor the nanopocket properties to the demands of a specific application.

Envisioned applications of these types of nanostructures and nanoparticles span a wide field from medicine [46] to electronics in general [47], particularly in photovoltaics [12], which can be attributed to the high level of customizability of the nanostructure properties. In the following, we will give some examples of possible applications.

Biomolecules can be captured by the nanopockets once a specific bio-functionalization is achieved. Gold is a very widely applied material substrate for specific biofunctionalization, which is often accomplished via gold-thiol linker chemistry [46]. When specifically targeting the biofunctionalization inside the nanopocket structure, i.e., the hollow cavity, we propose to use one of the following strategies. First, one can passivate the outer gold surface of the nanopockets when they are still bound to the substrate before doing the substrate transfer (this corresponds to the Figure 1g and Figure 3). After substrate transfer, only the gold layer inside the cavity will be reactive to thiol chemistry and can, therefore, be specifically functionalized to capture a chosen target molecule. Second, one can choose a layer configuration that results in nanopockets with a gold layer that is present only inside the cavity and another material for the surface outside the cavity (no symmetric composition of the single materials). By this, different binding chemistry approaches can be employed for the different materials and will result in a functionalization of the cavity only.

Regarding biomedical applications, the nanoparticles presented here could be beneficial for the detection of specific analyte molecules in a sample solution by making use of the optical [48,49] and magnetic properties of nanoparticles [50]. Additionally, the nanopockets can be very interesting for biomedical imaging methods. Gold nanoparticles are already widely employed for biological optical imaging methods, and nanoparticles with tailored physical properties are of great interest within this field of research [41,51]. We are also optimistic that the nanostructures and nanopockets might be very useful for surface-enhanced Raman spectroscopy techniques as these also make use of the electric field enhancement by nanoparticles and nanostructured surfaces [52,53,54,55]. Furthermore, one could benefit from the cavities of the nanopockets to fabricate nanoparticles for drug delivery [56] as biomolecules could be captured in the pockets.

The advantage of the nanopocket structure over simple gold spheres is the more complex geometry and the fact that the nanopockets show ferromagnetic behavior, which can be employed to manipulate the orientation of the nanopockets by external magnetic fields. Gold spheres will not show a dependence of their optical characteristics upon the polarization direction of the light employed, while the nanopockets do so as shown by optical transmittance measurements given in Figure 8. Manipulating their orientation by external fields and optical properties that depend on this orientation offers additional functionalities in comparison to spherical nanoparticles that can be exploited for biological applications relying on optical signal readout.

Alternative applications can be found in photovoltaics, photocatalysis and photochemistry. Here, surface plasmon resonance phenomena of various nanostructures (e.g., arrays of nanoholes [57]) are applied to trigger chemical reactions under light illumination [42]. Widely known examples include the use of combined metal and titanium dioxide nanostructures [43,58]. Finally, similar periodic array structures as those shown here are employed to realize nanolasing devices [59,60].

In conclusion, the fabrication technique of the nanostructured surfaces as well as of the multifunctional nanopockets presented here provides a powerful tool for nanofabrication due to its simplicity, flexibility and scalability. A wide range of different nanomaterials with tailor-made physical properties can be realized. Thus, many different applications may greatly benefit from the fabrication technique and its versatility.

## Figures and Tables

**Table 1:** Comparison of the experimentally measured and the simulated minima peak positions of the optical transmittance of the nanopocket array on a transparent substrate. The polarization of the incident light beam is aligned parallel to the long particle ellipse axis (left table half) as well as parallel to the short particle ellipse axis (right table half).

Polarization Parallel to Long Particle Ellipse Axis		Polarization Parallel to Short Particle Ellipse Axis	
Experiment	Simulation	Experiment	Simulation
531 nm	525 nm	652 nm	599 nm
771 nm ^1^	695 nm	727 nm	768 nm
974 nm	938 nm	907 nm	885 nm ^1^

^1^ inflection point.
